# Childhood stress impairs social function through AVP-dependent mechanisms

**DOI:** 10.1038/s41398-019-0678-0

**Published:** 2019-12-09

**Authors:** Nichola M. Brydges, Jessica Hall, Caroline Best, Lowenna Rule, Holly Watkin, Amanda J. Drake, Catrin Lewis, Kerrie L. Thomas, Jeremy Hall

**Affiliations:** 1 Neuroscience and Mental Health Research Institute, Cardiff University, Hadyn Ellis Building, Maindy Road, Cardiff, CF24 4HQ UK; 2 National Centre for Mental Health, Cardiff University, Hadyn Ellis Building, Maindy Road, Cardiff, CF24 4HQ UK; 3 University/BHF Centre for Cardiovascular Science, University of Edinburgh, Edinburgh, UK; 4 School of Biosciences, Cardiff University, Museum Avenue, Cardiff, CF10 3AX UK; 5 MRC Centre for Neuropsychiatric Genetics and Genomics, Cardiff University, Hadyn Ellis Building, Maindy Road, Cardiff, CF24 4HQ UK

**Keywords:** Molecular neuroscience, Genetics

## Abstract

Impaired social function is a core feature of many psychiatric illnesses. Adverse experiences during childhood increase risk for mental illness, however it is currently unclear whether stress early in life plays a direct role in the development of social difficulties. Using a rat model of pre-pubertal stress (PPS), we investigated effects on social behaviour, oxytocin and arginine vasopressin (AVP) in the periphery (plasma) and centrally in the paraventricular and supraoptic hypothalamic nuclei. We also explored social performance and AVP expression (plasma) in participants with borderline personality disorder (BPD) who experienced a high incidence of childhood stress. Social behaviour was impaired and AVP expression increased in animals experiencing PPS and participants with BPD. Behavioural deficits in animals were rescued through administration of the AVPR1a antagonist Relcovaptan (SR49059). AVP levels and recognition of negative emotions were significantly correlated in BPD participants only. In conclusion, early life stress plays a role in the precipitation of social dysfunction, and AVP mediates at least part of this effect.

## Introduction

Altered social function is a core component of several adult psychiatric illnesses. For example, depression and schizophrenia are commonly associated with social withdrawal, while major personality disorders can be associated with unstable social relationships (borderline personality disorder, BPD) or aggressive interactions (antisocial personality disorder)^1–4^. Impairments in social behaviour can also be a key determinant of functional outcome in these conditions^5–7^. However, relatively little is known about the causes of impaired social function in psychiatric conditions and their relationship to important aetiological factors associated with psychiatric disorders such as developmental stressors.

Early life stress has been shown to impact on social behaviour and functioning in both animal and human studies^8–13^. In rodent models, social stressors such as social isolation and social defeat during development negatively impact social function in adulthood, and interestingly non-social, physical stressors are also capable of eliciting substantial changes in social function^12,14,15^. The exact nature and timing of stress often influences later outcomes. The majority of studies have focussed on stressors early in development such as maternal separation, but less is known about the effects of stress in the post-weaning, pre-pubertal phase, a time-point suggested as more akin to human childhood^16–18^. During the pre-pubertal and adolescent phases the limbic system and prefrontal cortex are undergoing significant maturation. These areas are intimately involved in social function and extremely stress reactive due to high densities of corticosteroid receptors^19^. Therefore, stress during this time is predicted to have significant negative impacts on social function. Studies to date have revealed that non-social stress during the peri-pubertal phase results in enhanced aggression, and physical, pre-pubertal stressors alter social interaction in adulthood^12,15,20–25^. In humans, adverse early life experiences have been strongly associated with a range of later difficulties in social interaction in longitudinal and cross-sectional studies^8,9,26,27^. In particular, childhood and adolescent stressors have been linked to later social anxiety, withdrawal and aggression, although there is substantial variation in outcomes and not all exposed individuals are affected^28–32^. There is also substantive evidence that childhood adversity is associated with an increased risk for psychiatric disorders in which altered social interaction is a prominent feature, including social anxiety, depression and personality disorders^33–35^. A particularly strong association between developmental adversity and later illness is seen in the case of BPD in which impaired social interactions are a core feature of the presentation^34,36–40^. BPD is also characterised by an unstable sense of self, interpersonal sensitivity, impulsivity, cognitive disturbances (e.g., verbal comprehension, visual attention, working memory and processing speed) and intense, volatile emotions^41–43^. There is currently a lack of valid animal models that translate the key features of BPD, but the role of early life stress has recently been highlighted as a contributing aetiological factor that is amenable to study in animal models^44^. Indeed, certain features of BPD are paralleled by behavioural disturbances following peri-pubertal stress in animal models. For example, rodents exposed to physical or social stress during this period exhibit altered impulsivity and compulsivity and impaired working memory^45–47^.

The mechanisms through which early life stressors impact later social behaviour are still largely unknown. The evolutionarily conserved social neuropeptides oxytocin (OXT) and arginine vasopressin (AVP) are known to play an important role in social behaviour across species^48–52^. AVP and OXT are evolutionarily ancient nonapeptides found in various guises throughout the animal kingdom^53^. In mammals, they are predominantly manufactured in the paraventricular and supraoptic nuclei of the hypothalamus, and released into peripheral circulation via the pituitary gland^48^. OXT and AVP play a wide variety of roles in social behaviour in animals, ranging from inter-male and maternal aggression, conspecific affiliation, social cognition and sexual behaviour^48,50,51^. In humans, intranasal administration of both OXT and AVP influence emotion processing and social cognition, and genetic and functional neuroimaging studies demonstrate a role for OXT and AVP on social behaviour and related limbic brain circuitry^54,55^.

Maternal separation in rodents has been shown to increase AVP levels specifically in the paraventricular nucleus (PVN) and alter OXT levels and OXT/AVP receptor binding in an age and sex specific manner in the offspring^17,56–58^. However, less is known about the effects of stressors applied pre-pubertally, a period which may be more analogous to human childhood, on social peptide regulation. In humans, experience of childhood maltreatment has been associated with decreased levels of OXT in women and men, but the effects of early life stress on AVP levels are less well known^59^.

Here, we report three experiments investigating the relationship between early life stress, AVP/OXT and social behaviour. The aim of experiment 1 was to investigate the effects of pre-pubertal stress (PPS) on social behaviour and levels of OXT and AVP in adult animals. Experiment 2 sought to determine whether changes in social behaviour resulting from PPS could be reversed thorough AVP receptor antagonism, using the AVPR1a antagonist Relcovaptan (SR49059). The third and final experiment assessed peripheral levels of AVP in patients with BPD (a group with a high incidence of childhood adversity) and controls and related these to social behaviour.

## Materials and methods

### Experiments 1 and 2

#### Subjects

Female and male Lister Hooded rats were bred in house from adult pairs (Charles River) at Cardiff University. Litters were weaned on postnatal day (PND) 21 and housed in same sex cages (32 × 50 × 21 cm) with littermates. Cages were lined with wood shavings, a cardboard tube and wooden stick were provided as enrichment, light was maintained on a 12:12-h light/dark cycle and food and water were provided ad libitum. All animal experimental methods were carried out in accordance with relevant guidelines and regulations of the European regulations on animal experimentation (Directive 2010/63/EU) and the UK Home Office Animals (Scientific Procedures) Act 1986. All experimental protocols were approved by the local ethical review body (AWERB) of Cardiff University. Eighty rats (male: 20 control, 20 PPS; female: 20 control, 20 PPS) were used for social testing in experiment 1, and one hundred and forty four (female: 22 control and vehicle, 24 control and Relcovaptan, 20 PPS and vehicle, 30 PPS and Relcovaptan; male: 12 control and vehicle, 14 control and Relcovaptan, 10 PPS and vehicle, 12 PPS and Relcovaptan) in experiment 2. In a separate cohort of animals (not subjected to behavioural testing), forty rats (male: 12 control, 10 PPS; female: 8 control, 10 PPS) were used for plasma collection (peripheral AVP), and a further 44 rats (male: 10 control, 13 PPS; female: 10 control, 11 PPS) for immunohistochemistry (central AVP).

#### Pre-pubertal stress

PPS was given to half of the litters on PND 25–27. This protocol has been described previously^60–62^, and was originally described by Jacobson-Pick and Richter-Levin^63^. Briefly, animals were given a 10 min swim stress in an opaque swimming tank (25 cm high, 34 cm diameter), 12 L capacity filled with 6 L of 25 ± 1 °C water on PND 25, 3 sessions of 30 min restraint stress (separated by 30 min breaks in the home cage) in plastic restraint tubes (15 cm length, 5 cm diameter) on PND 26, and three 30 min elevated platform exposures (separated by 60 min breaks in the home cage) on elevated platforms (15 × 15 cm, 115 cm high) on PND 27. Stressors took place in a designated room, separate from the holding room. After PPS, animals were returned to their home cages and holding rooms and left undisturbed (aside from cage cleaning) until early adulthood (PND 60–67). Litters were randomly allocated to experimental groups (PPS or control) based on order of birth. A minimum of five litters per group were used to minimise the effects of pseudo-replication, and litter of origin was accounted for in all statistical analyses.

#### AVPR1a antagonist

Relcovaptan (AVPR1a antagonist SR49059, Axon Medchem BV, The Netherlands) was dissolved in 15% dimethyl sulfoxide (DMSO) and 2% Tween 80 in 0.9% saline and administered intraperitoneally at a volume of 2 ml/kg. The dose selected was 1 mg/kg, as this dose reliably inhibits prosocial and autonomic effects of peripherally administered AVP^64,65^. Vehicle was 15% DMSO and 2% Tween 80 in 0.9% saline.

#### Social testing

Testing took place in a clear acrylic tank (65 cm × 65 cm × 40 cm high) placed on the floor in the centre of a dimly lit room (30 lux). Interactions were filmed from above, and a microphone was suspended above the tank, connected to Avisoft SASLab Pro (avisoft bioacoustics, Germany) to capture ultrasonic vocalisations, which are frequently emitted during murine social encounters. Videos were then watched back by an observer blind to group, and a range of common social behaviours were analysed, as well as latency to initial contact, duration of each contact, number of contacts and total contact time. Specific behaviours recorded were divided into two categories, ‘aggressive’ consisting of boxing, biting, fighting, mounting, pinning (one rat rolls onto back, and other pins from above), nose off (rats stand immobile facing one another with all four paws on the ground, a defensive strategy), run away, crawl over (one rat crawls over another, often marking with urine), head under body of the other animal, and ‘benign or friendly’ consisting of allogrooming, sniffing and following. Recorded vocalisations were analysed with Avisoft SASLab Pro programme. Very few 22 kHz vocalisations were produced, so all analyses are based on 50 kHz vocalisations. In rats, 22 kHz vocalisations are most often emitted in aversive situations (e.g., presence of predators, pain), whereas 50 kHz reflect more positive affective states (e.g., during social contact, mating, in response to drugs of abuse or food)^66^.

Three hours before testing, animals were single housed in their holding room, to increase the desire for social contact. One hour before testing they were transferred to the testing room, to habituate them to this environment. In experiment 2 only, half of the animals from each group were given 1 mg/kg Relcovaptan via intraperitoneal injection 30 min before testing, the remaining half were administered vehicle only. During testing, two stranger animals were placed into opposite sides of the arena, facing the wall, and allowed to freely interact for 15 min. After this time they were returned to their home cages. The arena was cleaned with ethanol wipes between pairs of animals. Animals were tested in same-sex pairs, with each member of a given pair originating from the same group and treatment condition (PPS or control, vehicle or Relcovaptan) but different litters, so the animals had not previously met. Each pair was treated as one experimental unit for behavioural analysis.

#### Tissue and plasma collection

##### Experiment 1

For plasma AVP and OXT analyses, animals were sacrificed at PND 60 using a rising concentration of CO_2_, decapitated and trunk blood was collected using EDTA microvette collection tubes (Sarstedt, Germany). Blood was spun at 1500 × *g* for 10 min, plasma was removed and stored at −20 °C. For immunohistochemistry, animals were killed at PND 60 by transcardial perfusion with 0.01 M phosphate-buffered saline and 4% paraformadelhyde (PFA) under anaesthesia for immunohistochemical analysis of AVP in the supraoptic and paraventricular nuclei. Brains were left in PFA overnight (4 °C), then transferred to 30% sucrose solution for cryoprotection. Coronal 30 µm sections were cut through the entire hypothalamic extent on a freezing microtome (Leica RM2245) and placed into a solution of cryoprotectant for storage at −20 °C until immunohistochemical analysis.

##### Experiment 2

After social testing animals were culled (half of the animals were culled 20 min after testing (direct), the rest 1 week later (delayed)) and trunk blood samples taken as in *experiment 1*.

### ELISA assays

For experiment 1, rat plasma samples were assayed untreated. For experiment 2 and all human samples, plasma samples were dried down by adding 2:1 ice-cold acetone:plasma, centrifuging at 3000 × *g* for 20 min, transferring the supernatant, adding 5× supernatant volume of ice-cold petroleum ether to the supernatant, centrifuging at 3000 × *g* for 10 min, discarding the top ether layer and drying the remaining aqueous layer under nitrogen gas. This remaining pellet was reconstituted with 250 µl of assay buffer and used for ELISA analysis. Arg^8^-vasopressin and OXT (Enzo Life Sciences, UK) ELISAs were conducted according to the manufacturer’s instructions.

### Immunohistochemistry

Three sections per animal per region (supraoptic nucleus (SON) and PVN) were stained for AVP. These were matched for bregma between different animals. Sections were washed between each step for 3 × 5 min in 0.01 M Tris-buffered saline (TBS, pH 7.4) and all steps were carried out at room temperature unless otherwise specified. Sections were blocked with BLOXALL (Vector laboratories, UK) for 10 min, then blocking solution (2% goat serum, 0.3% Triton-X in 0.01 M TBS) for 60 min, rabbit anti-AVP (1:7500 in blocking solution, AB1565, Millipore, UK) for 24 h followed by biotinylated goat-anti-rabbit (3 µg/ml in blocking solution) for 45 min, then 30 min in VECTASTAIN ABC reagent (avidin-biotinylated horseradish peroxidase complex, Vector Laboratories, UK), before developing in diaminobenzidine solution (Vector Laboratories, UK) for 5 min. Washed sections were mounted onto glass microscope slides and coverslipped with Vectamount (Vector Laboratories, UK). Slides were then imaged at 20× using an Axio Scan Z1 (Zeiss). The optical density of AVP immunoreactive cells in the supraoptic and paraventricular nuclei were quantified as grey density per area minus background in digitised images using Zen Blue software (Zeiss).

### Data analysis

Data were analysed using generalised linear models in JMP (statistical software, SAS Institute, Cary, NC, USA). Group (control or PPS), sex, Relcovaptan/vehicle (experiment 2 only), direct or delayed sacrifice (experiment 2 only) and all interactions were fitted as factors, and latency to contact, total contact time, average contact duration, specific behaviour (boxing, biting, fighting, mounting, pinning, nose offs, run away, crawl over, allogrooming and following), number and duration of vocalisations, AVP and OXT were fitted as responses. Spearman’s Rho was used to assess correlations between behavioural and AVP measures. Unlike AVP measures, it was not possible to obtain individual social data for each animal, as animals were tested in pairs, so the behavioural data for the pair was used in correlational analyses. *P* was adjusted to 0.004 to account for multiple comparisons.

## Experiment 3

### Participants

A total of 20 people with BPD were recruited from the National Centre for Mental Health (NCMH, Welsh Government funded research centre) participant pool database. Patients with BPD were chosen as this condition is typically associated with very high levels of childhood trauma^67^. A total of 18 healthy age and sex matched controls with no psychiatric disorder were recruited from the community. Exclusion criteria for all participants included current substance dependence, neurological illness, psychotic disorder diagnosis (bipolar I or schizophrenia) and pregnancy, and additionally no psychiatric disorder for control participants. The BPD group consisted of 16 females and 4 males, mean age 43.4 (range: 25–71), controls 14 females and 4 males, mean age 38.4 (range: 20–64). In the BPD group, 11 were being treated with antipsychotic medication and 13 were being treated with antidepressant medication. BPD diagnosis was confirmed in the BPD group and excluded in the controls using the SCID-II (Structured Clinical Interview for DSM Disorders II). Current symptoms of depression were rated using the Hamilton Rating Scale for Depression (HADS). Participants also completed the Childhood Trauma Questionnaire (CTQ), a self-report measure of incidence and severity of childhood trauma consisting of 28 statements relating to one of five subscales of neglect (physical and emotional) or abuse (emotional, physical and sexual). The study was approved by the NHS research ethics committee, and all participants gave informed, written consent, had the opportunity to discuss the study and understood they were free to withdraw at any point.

### Social testing

Participants were given the Ekman 60 faces task, which assess overall emotion recognition performance and identification of basic emotions. During testing, 60 different faces were presented one by one on a computer screen for 3 s, depicting the faces of actors portraying one of six basic emotions (fear, disgust, anger, sadness, happiness and surprise). Ten incidents of each emotion were portrayed during the task. Participants were given as long as needed to identify the emotion. The response choices were shown on the screen, and participants made their selection using a computer mouse. Responses were recorded automatically. Faces were selected from the Ekman and Friesen series of Pictures of Facial Affect^68^, a widely used and validated series of photographs in facial expression research.

### Sample collection and analysis

Participants were visited in their own homes. Blood samples were collected from participants before questionnaire administration and social testing. In total, blood samples were obtained from 17 BPD and 15 controls. Samples were placed in cool bags containing reusable freezer blocks and transferred to the laboratory where they were centrifuged at 16,000 × *g* for 15 min at 4 °C. The cell-free supernatant plasma fraction was removed and transferred to −80 °C storage until analysis. AVP was analysed in plasma following the same protocol as for animal samples, using human Arg^8^-vasopressin ELISA kit (Enzo Life Sciences, UK). Copeptin was analysed using human copeptin ELISA (Novus Biologicals, UK), according to manufacturer’s instructions.

### Data analysis

Data were analysed using generalised linear models or Mann–Whitney *U* tests in JMP (statistical software, SAS Institute, Cary, NC, USA). Data were checked for normality and homogeneity of variance and transformed to fit these assumptions when necessary. If data could not be transformed, non-parametric tests were used. For AVP and copeptin, group was fitted as a factor with AVP/copeptin as a response. For the Ekman task, group (control or BPD), emotion (fear, disgust, anger, sadness, happiness and surprise) and group*emotion were fitted as factors, and number of correct responses as the response. Subject was nested within group and fitted as a random factor to account for repeated measures. Due to non-normal, non-homogenous data, Mann–Whitney *U* tests were used to analyse responses to the HADS and CTQ questionnaires. Spearman’s Rho was used to assess correlations between (i) EKMAN and AVP measures, (ii) CTQ and AVP measures and (iii) CTQ and EKMAN measures. *P* values were adjusted using the Bonferroni correction to account for multiple comparisons.

## Results

### Experiment 1

**Fig. 1:**
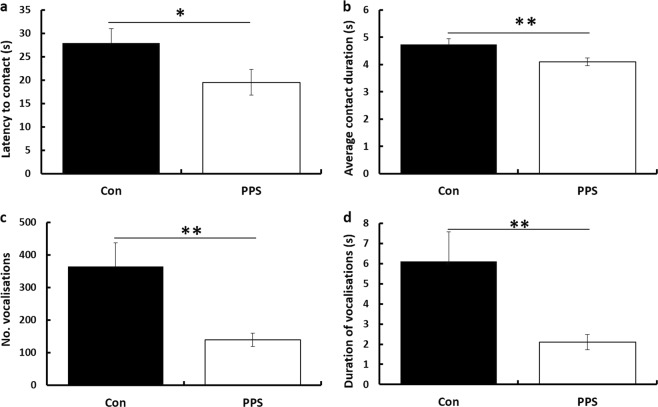
Social behaviour after PPS. PPS animals were **a** faster to initiate contact, **b** displayed shorter duration of contacts, **c** emitted fewer vocalisations which were **d** of shorter duration. *p < 0.05, **p < 0.01. Con control, PPS pre-pubertal stress. Error bars represent 1 S.E.

In experiment 1 we investigated the effects of PPS on social behaviour and AVP expression in adult rats. PPS decreased latency to initial social contact (*F*_1,35_ = 4.27, *p* = 0.046), decreased duration of each individual contact bout (*F*_1,35_ = 7.68, *p* = 0.009) and decreased number (*F*_1,36_ = 10.17, *p* = 0.003) and duration (*F*_1,36_ = 7.78, *p* = 0.008) of positively valanced ultrasonic vocalisations (50 kHz), and shortened maximum duration of vocalisations (*F*_1,36_ = 4.13, *p* = 0.049) in males and females (Fig. 1a–d). No biting or fighting were observed, and only nose-offs, sniffing, crawl overs and follows occurred frequently, with other behaviours rarely observed. There was no effect of PPS on the total number of contacts made (*F*_1,35_ = 2.25, *p* = 0.14), total contact time (*F*_1,35_ = 1.27, *p* = 0.27) or time engaged in any specific behaviour (boxing, nose-offs, mounts, pinning, run away crawl over, head under body, allogrooming or following, see Supplementary Table 1 for full statistical results). There were also sex differences in social behaviour, with males displaying increased duration of individual contact bouts (*F*_1,35_ = 7.35, *p* = 0.01), greater total contact time (*F*_1,35_ = 5.31, *p* = 0.03), fewer incidents of running away (*F*_1,35_ = 5.53, *p* = 0.02) and more crawl overs (*F*_1,35_ = 5.23, *p* = 0.03), and females generating more ultrasonic vocalisations (*F*_1,36_ = 5.73, *p* = 0.02) for a longer duration (*F*_1,35_ = 4.73, *p* = 0.04) and greater maximum duration (*F*_1,35_ = 10.99, *p* = 0.02).

**Fig. 2:**
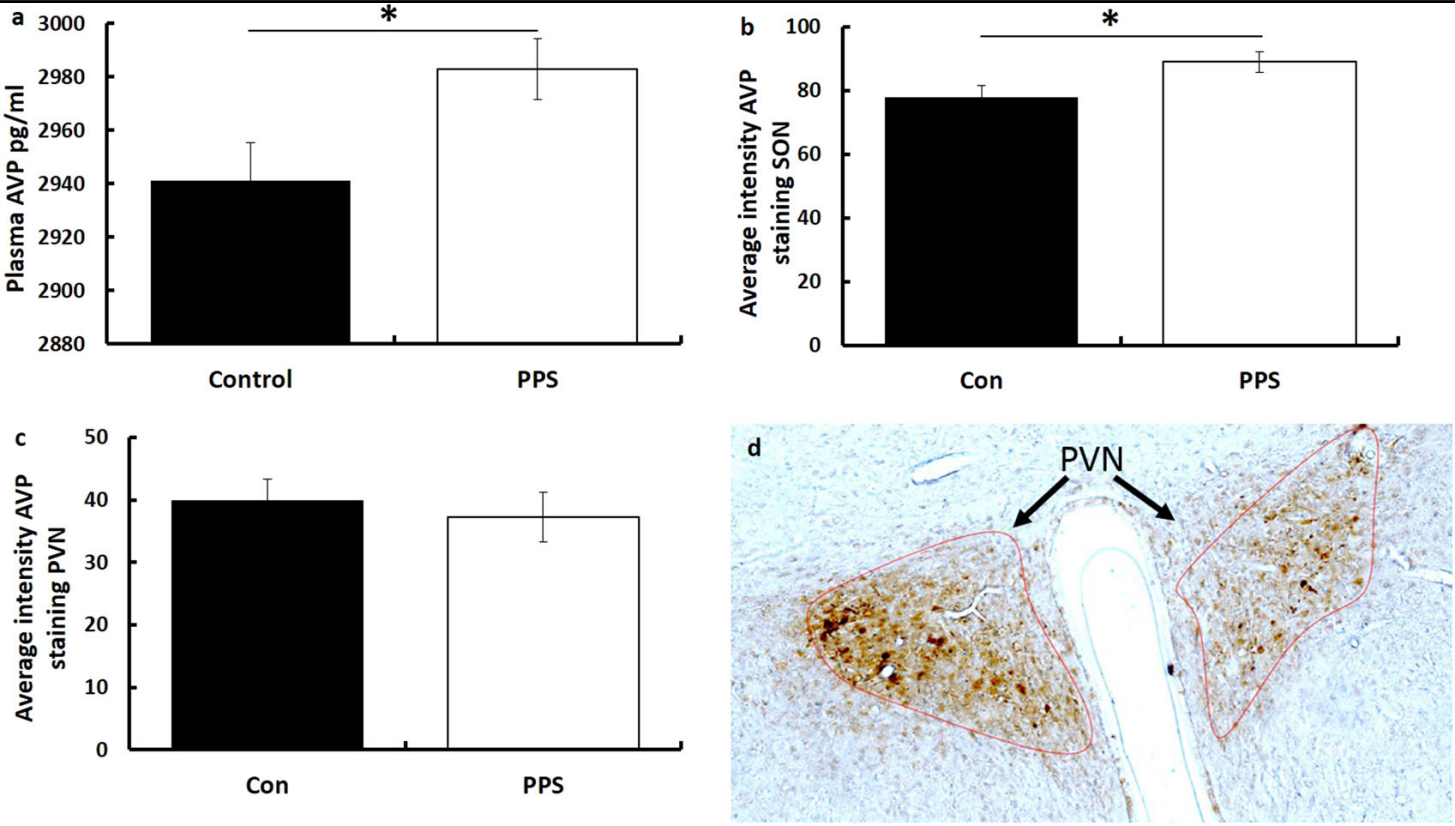
AVP levels after PPS. Following PPS, levels of AVP were elevated in **a** plasma and **b** supraoptic nucleus but not **c** paraventricular nucleus. **d** A representative immunohistochemistry image. *p < 0.05. Error bars represent 1 S.E.

In separate cohorts (not behaviourally tested) PPS resulted in higher AVP levels in plasma (*F*_1,36_ = 4.29, *p* = 0.04, Fig. 2a) and centrally in the supraoptic (*F*_1,39_ = 5.31, *p* = 0.03, Fig. 2b) but not paraventricular (*F*_1,36_ = 0.23, *P* = 0.64, Fig. 2c, d) nucleus of the hypothalamus as assessed by immunohistochemistry. PPS did not influence plasma levels of OXT (*F*_1,36_ = 0.01, *p* = 0.91). There were no effects of sex on plasma AVP (*F*_1,36_ = 1.42, *p* = 0.24) or AVP in the supraoptic (*F*_1,36_ = 1.33, *p* = 0.26) or paraventricular (*F*_1,36_ = 0.09, *p* = 0.76) nucleus.

### Experiment 2

**Fig. 3:**
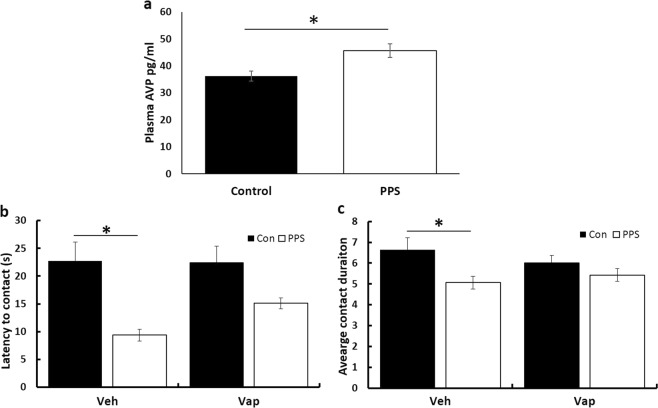
Effects of Relcovaptan on social behaviour after PPS. **a** Following PPS plasma levels of AVP were again elevated. PPS animals were **b** faster to initiate contact, this was reversed by administration of Relcovaptan, and **c** displayed shorter duration of contacts, which was again reversed by Relcovaptan. *p < 0.05. Error bars represent 1 S.E.

In experiment 2, we investigated the ability of an AVPR1a antagonist, Relcovaptan, to rescue behavioural changes induced through PPS in our animal model. As in experiment 1, PPS significantly increased plasma levels of AVP in these animals (*F*_1,139_ = 6.95, *p* = 0.01, Fig. 3a). Replicating our findings in experiment 1, PPS reduced latency to initial contact and decreased duration of each individual contact during a social test, but administration of Relcovaptan substantially reduced this effect (group*treatment: latency: *F*_1,64_ = 4.9, *p* = 0.004, contact: *F*_1,64_ = 3.1, *p* = 0.03, data box-cox transformed, Fig. 3b, c). For full statistical results please see Supplementary Table 2a, and for means and standard errors Supplementary Table 2b. There was no correlation between AVP and any behavioural measure in the social task (see Supplementary Table 2a for full statistical results).

### Experiment 3

**Fig. 4:**
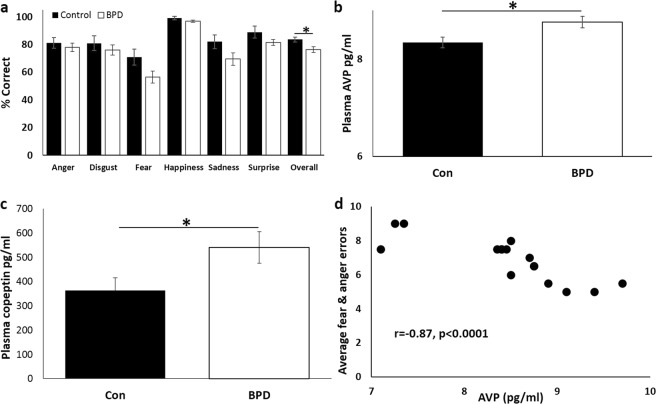
Social behaviour and AVP levels in BPD and controls. BPD participants were **a** worse at recognising emotion in the EKMAN task, **b** had higher plasma levels of AVP and **c** copeptin, and **d** within the BPD group only, higher AVP correlated with enhanced detection of negative emotions (fear and anger). *p < 0.05. Error bars represent 1 S.E.

We next investigated AVP levels and social cognition (assessed using the EKMAN 60 faces task) in participants with BPD, a condition strongly associated with early life stress. There was no significant difference in age or sex between control and BPD groups (*F*_1,36_ = 1.1, *p* = 0.3). Participants with BPD scored higher on the HADS (*S* = 192, *p* < 0.001) and CTQ across all abuse domains (emotional abuse: *S* = 181.5, *p* < 0.0001; physical abuse: *S* = 231, *p* < 0.0001; sexual abuse: *S* = 261, *p* < 0.0007; emotional neglect: *S* = 196, *p* < 0.0001; physical neglect: *S* = 218, *p* < 0.0001). Overall, BPD participants were worse at recognising emotion in the EKMAN task (*F*_1,36_ = 7.26, *p* = 0.01, Fig. 4a), replicating some previous findings but contrasting others^69–71^. This was not specific to any emotion, as demonstrated by the lack of group*emotion interaction (*F*_5,180_ = 0.83, *p* = 0.53). There was also a significant effect of emotion, overall participants were better at recognising happiness, and worse at recognising fear (*F*_5,180_ = 17.23, *p* < 0.0001). Plasma levels of AVP were higher in BPD compared to control participants (*F*_1,28_ = 5.66, *p* = 0.03, Fig. 4b), reflecting the higher peripheral AVP levels seen in the rat PPS model. Measurement of AVP has been suggested as problematic due to rapid clearance from circulation, instability in plasma and high platelet binding^72^. Therefore, we also measured plasma levels of copeptin, the c-terminal segment of the AVP precursor peptide, which has been suggested a more stable, surrogate marker of AVP release^73^. Copeptin was also elevated in the BPD patients (*F*_1,28_ = 4.36, *p* < 0.05, Fig. 4c). Within the BPD group only there was a negative correlation between plasma AVP and number of errors in recognising threat-related emotions, such that higher AVP corresponded with enhanced responsiveness to negative emotions (average of fear and anger, *r*_S_ = −0.87, *p* < 0.0001, Fig. 4d). Higher levels of emotional neglect were correlated with an increased ability to recognise anger (*r*_S_ = 0.7, *p* < 0.001) within the BPD group. There were no other correlations between AVP, emotion recognition and scores on the CTQ (see Supplementary Table 3 for full statistical report).

## Discussion

PPS resulted in altered social behaviour and elevated protein levels of AVP peripherally in plasma and centrally in the SON in rats. Relcovaptan, an AVPR1a antagonist, reversed behavioural changes induced by PPS. Similarly, individuals with BPD, a population with a high incidence of childhood adversity, had elevated plasma AVP and altered performance on tests of social emotion recognition. Furthermore, AVP levels correlated with increased sensitivity to negative emotional expressions in the BPD group. This suggests the AVP system is at least one mechanism through which early life stress can result in social difficulties in adulthood.

In our animal model, PPS resulted in a sustained increase in AVP levels both peripherally (blood plasma) and centrally (SON). AVP is mainly synthesised in the magnocellular neurons of the SON and PVN and parvocellular neurons of the PVN^74^. Parvocellular neurons project to the zona externa of the median eminence, and AVP originating from these neurons is suggested to be intimately involved in neuro-endocrine HPA axis function at rest and following acute and chronic stress^75,76^. In contrast, magnocellular neurons project through the zona interna of the median eminence to the posterior pituitary, where AVP is stored in axon terminals and upon stimulation, secreted into systemic blood circulation. This suggests that increased peripheral AVP in our PPS animals is a direct result of the observed upregulation in the magnocellular neurons of the SON. Previously, early life stress in the form of maternal separation increased AVP expression in the PVN of adult mice, but levels in the SON were unaffected^57^. It is unknown whether elevated PVN AVP would produce similar peripheral increases following early life stress as the previous maternal separation study did not examine peripheral AVP levels.

The differential effect of maternal separation and later juvenile stress on AVP in different nuclei of the hypothalamus could result from differences in the stress protocol (short-term physical stress vs. maternal separation) or the developmental time point (PND 25–27 vs. PND 1–10). This highlights the importance of timing—it is likely that different structures and processes are vulnerable to stressful perturbations at different time points during development. The nature of the stress is also likely to produce different effects in SON and PVN—in adult rats exposure to cold and heat stress increases AVP expression in the PVN, whereas SON AVP only responds to cold stress^77^. Furthermore, differences in the AVP system following early life stress may not be apparent until the system is challenged in a specific manner. AVP was increased in the SON and PVN of maternally separated adult rats and the SON of female prairie voles subjected to chronic social isolation only after experience of stressful intrasexual social, but not non-social, tests^78–80^.

In contrast to AVP, we found no changes in OXT following PPS. OXT is also produced in the SON and PVN and responds to certain early life stressors. In rodents, prenatal stress reduces OXT in the PVN, whereas maternal separation has no effect (although OXT receptor binding is altered)^13,56,81–83^. In humans, lower levels of OXT have been detected after severe childhood maltreatment in women, and after early life stress before the age of 12 but not during adolescence in men^84,85^. Conversely, less severe forms of childhood physical abuse result in increased OXT in adulthood^86^, again highlighting the likely importance of stressor nature and timing for later outcomes.

During a social interaction test, rats exposed to PPS displayed decreased latency to initial contact and decreased duration of individual contact bouts, and number and duration of positively valanced ultrasonic vocalisations were also reduced. Previous work has found that PPS decreases conspecific social interaction in adult males interacting with both adults (rats)^21,23^ and juveniles (mice)^20^ (but see ref. ^87^). In animals, AVP is important for social behaviour: disruption of AVP neurons impairs social memory specifically, and AVPR1a^−/−^ and AVPR1b^−/−^ mice demonstrate aberrant social recognition^88–90^. AVP infusion into the olfactory bulb and lateral septum facilitates social recognition memory in rats, and along with AVPR1a, AVP modulates aggression through several brain regions^51,91–94^. This raised the question of whether increased AVP following PPS in our animal model was directly responsible for altered social behaviour. We therefore investigated the ability of an AVPR1a antagonist, Relcovaptan (SR49059), to rescue behaviour following PPS. Social behaviour resulting from PPS was partially restored by administration of Relcovaptan, suggesting that AVP mediates some of the effects of PPS on adulthood social behaviour, and that this is a reversible phenomenon. Other neurobiological systems and receptors may also mediate the effects of PPS on later social behaviour. AVP can also exert effects through AVP receptor 1b and the OXT receptor to influence social behaviour, and future experiments should address the contribution of these receptor subtypes to the social phenotype observed following PPS. The involvement of other hormones with a known role in social behaviour, particularly those which are known to be responsive to early life stress (e.g., monoamines, corticotrophin releasing hormone and glucocorticoids)^12^ should similarly be investigated.

We next investigated whether AVP levels are also elevated in a psychiatric condition associated with high levels of childhood adversity, namely BPD. As with our animal model, we find that AVP protein was elevated in the plasma of BPD participants. Concordant with animal research, AVP and AVPR1a are strongly implicated in human social behaviour. AVPR1a promoter polymorphisms (particularly in RS3 microsatellite repeats) are associated with variation in prosocial behaviour, especially empathy and altruism, and polymorphisms in AVPR1a and AVPR1b have been shown to associate with social dysfunction in autism spectrum disorder^83,95–98^. In the present study, BPD participants demonstrated altered social behaviour. Specifically, they displayed decreased sensitivity to recognising facial expressions of emotion in the Ekman task when compared to controls. This supports previous findings of impaired social cognition in BPD^10,36,40,69,71^. We also find that higher AVP is correlated with higher sensitivity to detect fear and anger in this group, suggesting a role for AVP in the recognition of emotions directly related to threat perception in BPD. Interestingly, BPD are a group already known to display heightened attention to threat related social cues^41^. In agreement with our findings, in healthy individuals, some studies have found that intranasal administration of AVP enhances threat perception and detection of anger^99,100^. Others have found the opposite effect, with AVP administration decreasing recognition of negative emotions in men^101^. The precise effects may be affected by the particular group under study: intranasal AVP decreases anger detection in schizophrenic men, yet improves fear recognition in schizophrenic women^101^. Based on our current results it may be predicted that AVPR1a antagonism would further impair recognition of fear and anger in the BPD group, but we cannot predict the consequences for recognising other emotions. This would be an interesting area for future research.

We found no sex differences in response to PPS across any of our measures, with males and females being equally affected. There are well-known sex-differences in the AVP and OXT systems, for example the expression of AVP and AVPR1a are typically higher in males, and the administration of AVP and AVPR1a antagonists can produce differing effects in males and females^102^. Previous research in our laboratory has demonstrated striking sex differences in response to our PPS paradigm, for example males display impaired fear conditioning and altered hippocampal neurogenesis, whereas females are unaffected^103^. Conversely, PPS females show greater alterations in the hypothalamic–pituitary–adrenal axis^104^. This demonstrates that males and females display sex-based vulnerabilities and resiliencies to the lasting effects of PPS in certain domains, but are equally affected in others, highlighting the need to consider males and females in pre-clinical and clinical studies. In the social interaction task we found sex differences in behaviour regardless of treatment, with males spending more time in contact (increased average duration of contact and total contact time) and females vocalising more and performing more crawl overs and run-aways (see Supplementary Table 1).

This translational study provides evidence from across species for the importance of AVP in relation to early life stress and social behaviour. Of particular interest is the fact that despite the different stressor types administered in the animal model and experienced by the BPD cohort, both populations experienced increased AVP and altered social behaviour. The exact contribution and consequences of different stressor types to the precipitation of behavioural and biological alterations later in life are not fully understood, and should form the basis of future research. Our results demonstrate that the AVP system may be a useful target for ameliorating social difficulties in conditions associated with high rates of childhood trauma, such as BPD, and that peripheral AVP levels may represent a valuable biomarker for stratification of treatment in such groups. As a high rate of childhood adversity is found in other psychiatric populations, for example depression and anxiety, these would be valuable cohorts for future study. Future studies should also aim to measure broader domains of social function, to further align social measures between human and animal studies. Additional scales and self-reports of social interaction would be informative in human populations, and the response of PPS animals to aversive social situations such as tests of social dominance and resident-intruder paradigms would prove informative and enhance cross-species translation.

In conclusion, we find that experience of stressful events early in life is likely to play a role in the development of social abnormalities in adulthood, and alterations in the AVP system are at least partially responsible for this effect. AVPR1a is a promising target for intervention in disorders with a social phenotype, especially those produced thorough stressful early life experiences.

## Supplementary information

Supplementary table 1

Supplementary table 2

Supplementary table 3
