# Supplementary material for: Childhood stress impairs social function through AVP-dependent mechanisms
Source: Transl Psychiatry. 2019 Dec 9;9:330. doi: 10.1038/s41398-019-0678-0 (PMC6901493; doi:10.1038/s41398-019-0678-0)
Supplement: Supplementary file 1 — Supplementary table 1 [file 41398_2019_678_MOESM1_ESM.docx]

| **Experiment/Factor** | **Group** | **Sex** | **Group*sex** | **Mean** | | | **SE** | |
| --- | --- | --- | --- | --- | --- | --- | --- | --- |
| ***Experiment 1*** |  |  |  | **Control** | | **PPS** | **Control** | **PPS** |
| Latency to social contact (sec) | ***F_1,35_=4.27, p=0.046*** | F_1,35_=2.75, p=0.11 | F_1,35_=0.31, p=0.58 | **27.9** | **19.53** | | 3.16 | 2.77 |
| Average duration of contact (sec) | ***F_1,35_=7.68, p=0.009*** | ***F_1,35_=7.35, p=0.01*** | F_1,35_=0.01, p=0.92 | **4.74** | **4.1** | | 0.21 | 0.14 |
| Number of contacts | F_1,35_=2.25, p=0.14 | F_1,35_=0.02, p=0.89 | F_1,35_=0.06, p=0.81 | 54.55 | 59.05 | | 2.27 | 1.84 |
| Total contact time (sec) | F_1,35_=1.27, p=0.27 | ***F_1,35_=5.31, p=0.03*** | F_1,35_=0.08, p=0.77 | 255.7 | 239.32 | | 13.69 | 7.74 |
| Boxing | F_1,35_=2.06, p=0.16 | F_1,35_=0, p=1 | F_1,35_=0.52, p=0.48 | 0.25 | 0.05 | | 0.12 | 0.05 |
| Mounts | F_1,35_=0.21, p=0.65 | F_1,35_=1.36, p=0.25 | F_1,35_=0.48, p=0.49 | 0.7 | 1.05 | | 0.23 | 0.84 |
| Pins | F_1,35_=1.93, p=0.17 | F_1,35_=1.93, p=0.17 | F_1,35_=1.93, p=0.17 | 0.5 | 0 | | 0.36 | 0 |
| Nose-offs | F_1,35_=4.18, p=0.06 | F_1,35_=3.08, p=0.09 | F_1,35_=0.02, p=0.89 | 4.6 | 2.84 | | 0.74 | 0.41 |
| Run away | F_1,35_=0.45, p=0.51 | ***F_1,35_=5.53, p=0.02*** | F_1,35_=0.45, p=0.51 | 0.2 | 0.11 | | 0.12 | 0.07 |
| Crawl over | F_1,35_=0.0002, p=0.99 | ***F_1,35_=5.23, p=0.03*** | F_1,35_=0.49, p=0.49 | 12.1 | 12.21 | | 1.13 | 1.47 |
| Head under body | F_1,35_=0.24, p=0.63 | F_1,35_=0.58, p=0.45 | F_1,35_=0.02, p=0.89 | 0.9 | 0.74 | | 0.26 | 0.17 |
| Allogrooming | F_1,35_=0.34, p=0.56 | F_1,35_=2.3, p=0.14 | F_1,35_=0.34, p=0.56 | 0.2 | 0.32 | | 0.12 | 0.15 |
| Follow | F_1,35_=0.05, p=0.81 | F_1,35_=0.78, p=0.38 | F_1,35_=0.01, p=0.93 | 6.55 | 6.21 | | 1.14 | 0.63 |
| Number USV vocalisations | ***F_1,36_=10.17, p=0.003*** | ***F_1,36_=5.73, p=0.02*** | F_1,36_=1.93, p=0.17 | **364.9** | **139.35** | | 72.95 | 20.6 |
| Total duration USV vocalisations (sec) | ***F_1,36_=7.78, p=0.008*** | ***F_1,36_=4.73, p=0.04*** | F_1,36_=1.89, p=0.18 | **6.1** | **2.1** | | 1.47 | 0.38 |
| Maximum duration USV vocalisations (ms) | ***F_1,36_=4.13, p=0.049*** | ***F_1,36_=10.99, p=0.02*** | F_1,36_=1.74, p=0.2 | **86.33** | **72.86** | | 5.35 | 5.26 |
| AVP plasma | ***F_1,36_=4.29, p=0.04*** | F_1,36_=1.42, p=0.24 | F_1,36_=1.39, p=0.25 | **2941.31** | **2982.96** | | 13.92 | 11.45 |
| OXT plasma | F_1,36_=0.01, p=0.91 | F_1,36_=2.16, p=0.15 | F_1,36_=0.36, p=0.55 | 3151.15 | 3172.86 | | 54.02 | 82.06 |
| AVP supraoptic nucleus (intensity staining) | ***F_1,36_=5.31, p=0.03*** | F_1,36_=1.33, p=0.26 | F_1,36_=0.47, p=0.5 | **77.77** | **88.97** | | 3.67 | 3.26 |
| AVP paraventricular nucleus (intensity staining) | F_1,36_=0.23, p=0.64 | F_1,36_=0.09, p=0.76 | F_1,36_=0.0004, p=0.98 | 39.9 | 37.2 | | 3.35 | 4.02 |

Supplementary Table 1. Full statistical report of Experiment 1 and mean and standard errors (SE). Social behaviour testing, arginine vasopressin (AVP) and oxytocin (OXT) in plasma and AVP in the hypothalamus. Results shown in bold are significant.
