# Supplementary material for: Childhood stress impairs social function through AVP-dependent mechanisms
Source: Transl Psychiatry. 2019 Dec 9;9:330. doi: 10.1038/s41398-019-0678-0 (PMC6901493; doi:10.1038/s41398-019-0678-0)
Supplement: Supplementary file 2 — Supplementary table 2 [file 41398_2019_678_MOESM2_ESM.docx]

| **Experiment 2 - Behaviour** | **Group** | **Sex** | **Treatment** | **Group*sex** | **Group*Treatment** | **Sex*Treatment** | **Group*Sex***  **Treatment** |
| --- | --- | --- | --- | --- | --- | --- | --- |
| Latency to social contact (sec) | **F_1,64_=9.1, p=0.004** | F_1,64_=0.07, p=0.79 | F_1,64_=1.39, p=0.24 | F_1,64_=1.15, p=0.29 | **F_1,64_=4.9, p=0.004** | F_1,64_=0.07, p=0.79 | F_1,64_=0.03, p=0.87 |
| Average duration of contact (sec) | **F_1,64_=5.6, p=0.02** | F_1,64_=0.11, p=0.74 | F_1,64_=0.002, p=0.97 | F_1,64_=1.06, p=0.31 | **F_1,64_=3.1, p=0.03** | F_1,64_=0.03, p=0.87 | F_1,64_=0.23, p=0.63 |
| Number of contacts | F_1,64_=1.54, p=0.22 | F_1,64_=1.94, p=0.17 | F_1,64_=0.57, p=0.45 | F_1,64_=0.004, p=0.95 | F_1,64_=0.37, p=0.54 | F_1,64_=0.45, p=0.51 | F_1,64_=0.78, p=0.38 |
| Total contact time (sec) | F_1,64_=2.21, p=0.14 | F_1,64_=0.01, p=0.92 | F_1,64_=1.67, p=0.2 | F_1,64_=0.11, p=0.74 | F_1,64_=0.41, p=0.52 | F_1,64_=0.15, p=0.7 | F_1,64_=0.04, p=0.84 |
| Boxing | F_1,64_=1.2, p=0.28 | F_1,64_=2.48, p=0.12 | F_1,64_=0.22, p=0.64 | F_1,64_=0.67, p=0.42 | F_1,64_=0.23, p=0.63 | F_1,64_=0.44, p=0.51 | F_1,64_=0.91, p=0.35 |
| Mounts | F_1,64_=1.02, p=0.32 | F_1,64_=1.02, p=0.32 | F_1,64_=0.005, p=0.95 | F_1,64_=1.02, p=0.32 | F_1,64_=0.005, p=0.95 | F_1,64_=0.005, p=0.95 | F_1,64_=0.005, p=0.95 |
| Pins | F_1,64_=0.29, p=0.59 | F_1,64_=1.42, p=0.24 | F_1,64_=0.01, p=0.91 | F_1,64_=0.11, p=0.75 | F_1,64_=0.95, p=0.33 | F_1,64_=0.01, p=0.91 | F_1,64_=0.58, p=0.45 |
| Nose-offs | F_1,64_=0.04, p=0.85 | F_1,64_=1.39, p=0.24 | F_1,64_=0.04, p=0.84 | F_1,64_=0.11, p=0.74 | F_1,64_=0.59, p=0.44 | F_1,64_=1.02, p=0.32 | F_1,64_=0.29, p=0.59 |
| Run away | F_1,64_=0.59, p=0.44 | F_1,64_=0.8, p=0.37 | F_1,64_=0.8, p=0.37 | F_1,64_=0.59, p=0.44 | F_1,64_=0.59, p=0.44 | F_1,64_=0.8, p=0.37 | F_1,64_=0.59, p=0.44 |
| Crawl over | F_1,64_=1.54, p=0.22 | **F_1,64_=9.56, p=0.003** | F_1,64_=1.62, p=0.21 | F_1,64_=0.13, p=0.72 | F_1,64_=0.25, p=0.62 | F_1,64_=0.01, p=0.91 | F_1,64_=0.47, p=0.5 |
| Head under body | F_1,64_=0.09, p=0.77 | F_1,64_=3.16, p=0.08 | F_1,64_=0.06, p=0.81 | F_1,64_=0.1, p=0.75 | F_1,64_=1.65, p=0.2 | F_1,64_=0.76, p=0.39 | F_1,64_=0.44, p=0.51 |
| Allogrooming | F_1,64_=0.74, p=0.39 | F_1,64_=1.56, p=0.22 | F_1,64_=0.74, p=0.39 | F_1,64_=0.74, p=0.39 | F_1,64_=1.56, p=0.22 | F_1,64_=0.74, p=0.39 | F_1,64_=1.56, p=0.22 |
| Follow | F_1,64_=2.62, p=0.11 | F_1,64_=2.15, p=0.15 | F_1,64_=0.3, p=0.59 | F_1,64_=0, p=0.99 | F_1,64_=1.1, p=0.3 | F_1,64_=0.18, p=0.68 | F_1,64_=0.02, p=0.88 |
| **Experiment 2 – AVP plasma** | **Group** | **Sex** | **Treatment** | **Direct/Delay** | **Two-way interactions** | **Three-way interactions** | **Four-way interactions** |
| AVP plasma (pg/ml) | **F_1,139_=6.95, p=0.01** | **F_1,139_=7.1, p=0.01** | F_1,139_=0.09, p=0.77 | F_1,139_=0.07, p=0.79 | **Group*Treatment**: F_1,139_=2.99, p=0.09 **Group*Sex:**  F_1,139_=0.39, p=0.54 **Group*Direct/Delay:** F_1,139_=0.53, p=0.47 **Treatment*Sex:** F_1,139_=1.12, p=0.29 **Treatment*Direct/**  **Delay:** F_1,139_=1.09, p=0.3 **Direct/Delay*Sex:**  F_1,139_=1.01, p=0.32 | **Treatment*Sex***  **Group:** F_1,139_=0.22, p=0.64 **Group*Treatment*Direct/Delay:** F_1,139_=1.74, p=0.19  **Group*sex* Direct/Delay:** F_1,139_=0.007, p=0.93 **Treatment*Sex***  **Immediate/Delay:** F_1,139_=0.38, p=0.54 | **Group*Treatment***  **Direct/Delay***  **Sex:** F_1,139_=0.35, p=0.56 |
| ***Correlations*** | **Con, Vehicle** | **Control, Vaptan** | **PPS, Vehicle** | **PPS, Vaptan** |  | | |
| **AVP & Latency** | Di: r_S_=-0.44, p=0.08  De: r_S_=-0.13, p=0.61 | Di: r_S_=0.16, p=0.52  De: r_S_=-0.03, p=0.91 | Di: r_S_=0.1, p=0.73  De: r_S_=0.01, p=0.97 | Di: r_S_=-0.18, p=0.44  De: r_S_=0.07, p=0.78 |  |  |  |
| **AVP & No. contacts** | Di: r_S_=-0.01, p=0.98  De: r_S_=0.13, p=0.63 | Di: r_S_=0.17, p=0.5  De: r_S_=-0.11, p=0.66 | Di: r_S_=0.06, p=0.82  De: r_S_=-0.12, p=0.67 | Di: r_S_=-0.48, p=0.03  De: r_S_=0.56, p<0.01 |  |  |  |
| **AVP & total contact time** | Di: r_S_=-0.27, p=0.3  De: r_S_=0.05, p=0.86 | Di: r_S_=0.46, p=0.05  De: r_S_=-0.08, p=0.74 | Di: r_S_=-0.01, p=0.99  De: r_S_=-0.08, p=0.78 | Di: r_S_=0.21, p=0.37  De: r_S_=0.18, p=0.44 |  |  |  |
| **AVP & Mounts** | Di: r_S_=-0.05, p=0.85  De: No mounts | Di: r_S_=0.22, p=0.38  De: r_S_=0.04, p=0.86 | Di: r_S_= no mount  De: r_S_= no mounts | Di: r_S_= no mounts  De: r_S_= no mounts |  |  |  |
| **AVP & Pins** | Di: r_S_=0.25, p=0.33  De: r_S_=0.03, p=0.92 | Di: r_S_=0.39, p=0.1  De: r_S_=0.3, p=0.21 | Di: r_S_= no pins  De: r_S_= no pins | Di: r_S_=0.27, p=0.24  De: r_S_=0.23, p=0.32 |  |  |  |
| **AVP & Nose-offs** | Di: r_S_=-0.08, p=0.76  De: r_S_=-0.16, p=0.55 | Di: r_S_=0.21, p=0.38  De: r_S_=0.32, p=0.19 | Di: r_S_=-0.09, p=0.74  De: r_S_=0.21, p=0.47 | Di: r_S_=-0.04, p=0.87  De: r_S_=0.22, p=0.34 |  |  |  |
| **AVP & Crawl over** | Di: r_S_=0.02, p=0.94  De: r_S_=0.26, p=0.34 | Di: r_S_=0.08, p=0.75  De: r_S_=0.29, p=0.23 | Di: r_S_=0.03, p=0.92  De: r_S_=0.23, p=0.44 | Di: r_S_=0.06, p=0.78  De: r_S_=0.49, p=0.02 |  |  |  |
| **AVP & Head under** | Di: r_S_=no head under  De: r_S_=0.36, p=0.17 | Di: r_S_=0.3, p=0.21  De: r_S_=0.49, p=0.03 | Di: r_S_=-0.11, p=0.69  De: r_S_=0.53, p=0.05 | Di: r_S_=0.18, p=0.44  De: r_S_=-0.07, p=0.78 |  |  |  |
| **AVP & Allogroom** | Di: r_S_=0.03, p=0.92  De: r_S_=0.45, p=0.08 | Di: no allogrooming  De: no allogrooming | Di: r_S_= no allogroom  De: r_S_= no allogroom | Di: r_S_=-0.33, p=0.14  De: r_S_=0.02, p=0.94 |  |  |  |
| **AVP & Run away** | Di: r_S_=-0.05, p=0.85  De: No run aways | Di: no run aways  De: no run aways | Di: r_S_=0.43, p=0.11  De: no run away | Di: r_S_= no run away  De: r_S_= no run away |  |  |  |
| **AVP & Box** | Di: r_S_=-0.13, p=0.61  De: r_S_=-0.05, p=0.87 | Di: r_S_=0.19, p=0.44  De: r_S_=0.44, p=0.06 | Di: r_S_=-0.06, p=0.83  De: r_S_=0.32, p=0.26 | Di: r_S_=0.47, p=0.03  De: r_S_=0.33, p=0.14 |  |  |  |
| **AVP & Follow** | Di: r_S_=0.05, p=0.85  De: r_S_=0.28, p=0.3 | Di: r_S_=0.17, p=0.48  De: r_S_=-0.04, p=0.88 | Di: r_S_=0.06, p=0.84  De: r_S_=-0.16, p=0.6 | Di: r_S_=0.57, p=0.007  De: r_S_=0.08, p=0.73 |  |  |  |

Supplementary Table 2a. Full statistical report of Experiment 2. Social behaviour testing and arginine vasopressin (AVP) in blood and correlations. Results shown in bold are significant. Di = Direct sacrifice, De = Delay ‘baseline’ sacrifice. Missing correlations indicate that values for a behaviour are zero, and a correlation cannot be calculated. For correlations, p<0.004 was considered significant, after Bonferroni correction.

| **Experiment 2** | **Mean** | | | | **SE** | | | |
| --- | --- | --- | --- | --- | --- | --- | --- | --- |
|  | **Con, Vehicle** | **Control, Vaptan** | **PPS, Vehicle** | **PPS, Vaptan** | **Con, Vehicle** | **Control, Vaptan** | **PPS, Vehicle** | **PPS, Vaptan** |
| Latency to social contact (sec) | 22.71 | 22.37 | 10.47 | 15.1 | 3.44 | 3 | 1.48 | 1.81 |
| Average duration of contact (sec) | 6.64 | 6 | 5.07 | 5.43 | 0.57 | 0.37 | 0.31 | 0.21 |
| Number of contacts | 44.76 | 47.95 | 48.4 | 50 | 2.14 | 1.78 | 3.05 | 2.06 |
| Total contact time (sec) | 269.18 | 280.68 | 231.07 | 266.43 | 15.63 | 13.188 | 18.17 | 13.31 |
| Boxing | 0.94 | 0.89 | 0.2 | 0.76 | 0.48 | 0.31 | 0.14 | 0.27 |
| Mounts | 0.23 | 0.26 | 0 | 0 | 0.24 | 0.26 | 0 | 0 |
| Pins | 0.7 | 0.21 | 0 | 0.48 | 0.59 | 0.92 | 0 | 0.33 |
| Nose-offs | 5.53 | 5.15 | 5.06 | 5.6 | 0.67 | 0.58 | 0.74 | 0.54 |
| Run away | 0.06 | 0 | 0.8 | 0 | 0.06 | 0 | 0.8 | 0 |
| Crawl over | 4.18 | 5.63 | 3.2 | 4.29 | 1.12 | 0.75 | 0.7 | 1.05 |
| Head under body | 0.06 | 0.37 | 0.33 | 0.19 | 0.06 | 0.22 | 0.16 | 0.11 |
| Allogrooming | 0.47 | 0 | 0 | 0.1 | 0.32 | 0 | 0 | 0.1 |
| Follow | 5.53 | 7.37 | 4.87 | 4.61 | 0.95 | 1.23 | 1 | 0.88 |
| AVP plasma | 33.19 | 38.89 | 48.22 | 44.12 | 2.66 | 2.57 | 3.97 | 3.25 |

Supplementary Table 2b. Mean and standard error (SE) values for behavioural and blood arginine vasopressin (AVP) analyses in Experiment 2.
