# Supplementary material for: Childhood stress impairs social function through AVP-dependent mechanisms
Source: Transl Psychiatry. 2019 Dec 9;9:330. doi: 10.1038/s41398-019-0678-0 (PMC6901493; doi:10.1038/s41398-019-0678-0)
Supplement: Supplementary file 3 — Supplementary table 3 [file 41398_2019_678_MOESM3_ESM.docx]

| **Experiment/Factor** | **Group** | **Mean** | | **SE** | |
| --- | --- | --- | --- | --- | --- |
| ***Experiment 3*** |  | Control | BPD | Control | BPD |
| **Age** | F_1,36_=1.1, p=0.3 | 38.33 | 43.3 | 3.87 | 2.83 |
| **HADS** | ***S=192, p<0.001*** | 6.11 | 25.2 | 0.9 | 2.07 |
| ***CTQ***  Emotional abuse  Physical abuse  Sexual abuse  Emotional neglect  Physical neglect | ***S=181.5, p<0.0001***  ***S=231, p<0.0001***  ***S=261, p<0.0007***  ***S=196, p<0.0001***  ***S=218, p<0.0001*** | 5.61  5.33  5  6.39  5.39 | 14.7  9.6  9.65  14.55  9.35 | 0.31  0.24  0  0.46  0.2 | 1.28  1.17  1.54  1.18  0.92 |
| ***EKMAN***  Group  Emotion  Group*emotion  ***Mean % & SE***  All emotions  Anger  Disgust  Fear  Happiness  Sadness  Surprise | F_1,36_=7.26, p=0.01  F_5,180_=17.23, p<0.0001  F_5,180_=0.83, p=0.53 | 83.7  81.1  80.6  70.6  99.9  81.7  89.4 | 76.4  78  76  56.5  97  69.5  81.5 | 1.6  3.1  3.6  4.3  0.8  4.6  2.1 | 2.1  3.9  5.2  5.8  1.3  4.9  4.2 |
| **AVP plasma (pg/ml)** | F_1,28_=5.66, p=0.03 | 8.38 | 8.76 | 0.1 | 0.12 |
| **Copeptin plasma (pg/ml)** | F_1,28_=4.36, p<0.05 | 363.33 | 541.29 | 51.76 | 65.24 |
| ***Correlations*** | **Control** | **BPD** | |  | |
| ***AVP & CTQ, adjusted p<0.008***  AVP & emotional abuse  AVP & physical abuse  AVP & sexual abuse  AVP & emotional neglect  AVP & physical neglect | r_S_=-0.59, p=0.03  r_S_=0.42, p=0.14  r_S_=0, p=1  r_S_=0.001, p=1  r_S_=0.21, p=0.47 | r_S_=0.09, p=0.72  r_S_=0.33, p=0.22  r_S_=-0.05, p=0.85  r_S_=-0.33, p=0.22  r_S_=0.2, p=0.45 | |  |  |
| ***AVP & emotion recognition, adjusted p<0.007***  AVP & anger  AVP & disgust  AVP & fear  AVP & happiness  AVP & sadness  AVP & surprise  AVP & average fear & anger  ***CTQ & emotion recognition, adjusted p<0.002***  Anger & Emotional abuse  Anger & Physical abuse  Anger & Sexual abuse  Anger & Emotional neglect  Anger & Physical neglect  Disgust & Emotional abuse  Disgust & Physical abuse  Disgust & Sexual abuse  Disgust & Emotional neglect  Disgust & Physical neglect  Fear & Emotional abuse  Fear & Physical abuse  Fear & Sexual abuse  Fear & Emotional neglect  Fear & Physical neglect  Happiness & Emotional abuse  Happiness & Physical abuse  Happiness & Sexual abuse  Happiness & Emotional neglect  Happiness & Physical neglect  Sadness & Emotional abuse  Sadness & Physical abuse  Sadness & Sexual abuse  Sadness & Emotional neglect  Sadness & Physical neglect  Surprise & Emotional abuse  Surprise & Physical abuse  Surprise & Sexual abuse  Surprise & Emotional neglect  Surprise & Physical neglect | r_S_=0.26, p=0.37  r_S_=0.07, p=0.81  r_S_=-0.2, p=0.48  r_S_=-0.23, p=0.43  r_S_=0.07, p=0.82  r_S_=0.36, p=0.21  r_S_=0.07, p=0.81  r_S_=-0.003, p=0.99  r_S_=0.1, p=0.7  r_S_=0, p=1  r_S_=-0.1, p=0.7  r_S_=-0.28, p=0.26  r_S_=-0.06, p=0.81  r_S_=0.36, p=0.14  r_S_=0, p=1  r_S_=-0.15, p=0.55  r_S_=0.35, p=0.15  r_S_=-0.05, p=0.84  r_S_=-0.21, p=0.4  r_S_=0, p=1  r_S_=-0.32, p=0.19  r_S_=-0.42, p=0.08  r_S_=0.22, p=0.39  r_S_=0.12, p=0.62  r_S_=0, p=1  r_S_=0.14, p=0.57  r_S_=-0.19, p=0.46  r_S_=-0.22, p=0.39  r_S_=-0.14, p=0.57  r_S_=0, p=1  r_S_=-0.53, p=0.02  r_S_=-0.36, p=0.14  r_S_=-0.3, p=0.22  r_S_=-0.22, p=0.38  r_S_=0, p=1  r_S_=-0.24, p=0.33  r_S_=0.14, p=0.57 | r_S_=-0.44, p=0.09  r_S_=-0.1, p=0.72  r_S_=-0.64, p=0.008  r_S_=0.33, p=0.21  r_S_=-0.03, p=0.91  r_S_=0.31, p=0.25  **r_S_=-0.87, p<0.0001**  r_S_=0.18, p=0.46  r_S_=0.08, p=0.74  r_S_=-0.21, p=0.37  **r_S_=0.7, p<0.001**  r_S_=0.29, p=0.22  r_S_=-0.58, p=0.007  r_S_=-0.54, p=0.01  r_S_=-0.27, p=0.24  r_S_=-0.18, p=0.44  r_S_=-0.52, p=0.02  r_S_=-0.15, p=0.53  r_S_=-0.1, p=0.68  r_S_=-0.21, p=0.38  r_S_=0.07, p=0.76  r_S_=-0.45, p=0.04  r_S_=0.03, p=0.92  r_S_=0.11, p=0.64  r_S_=0.42, p=0.06  r_S_=-0.26, p=0.27  r_S_=0.19, p=0.43  r_S_=0.3, p=0.2  r_S_=0.12, p=0.62  r_S_=0.35, p=0.13  r_S_=0.19, p=0.41  r_S_=0.04, p=0.87  r_S_=-0.33, p=0.15  r_S_=0.07, p=0.77  r_S_=0.008, p=0.97  r_S_=-0.15, p=0.52  r_S_=0.21, p=0.38 | |  |  |

Supplementary Table 3. Full statistical results from experiment 3. Childhood trauma questionnaire (CTQ), Ekman task,

arginine vasopressin (APV) and copeptin in plasma. Results shown in bold are significant.
